# Metamaterials of Auxetic Geometry for Seismic Energy Absorption

**DOI:** 10.3390/ma16155499

**Published:** 2023-08-07

**Authors:** Ahmed Abdalfatah Saddek, Tzu-Kang Lin, Wen-Kuei Chang, Chia-Han Chen, Kuo-Chun Chang

**Affiliations:** 1 Department of Civil Engineering, National Yang Ming Chiao Tung University, Hsinchu 300093, Taiwan; ahmedsaddek3@gmail.com (A.A.S.);; 2 National Center for Research on Earthquake Engineering, Taipei 106219, Taiwan; 3 Department of Civil Engineering, National Taiwan University, Taipei 106319, Taiwan; ciekuo@ntu.edu.tw

**Keywords:** auxetic, periodic structure, seismic metamaterial, auxetic geometry, bandgap

## Abstract

The propagation of earthquake energy occurs primarily through elastic waves. If the seismic force input to a structure can be directly reduced from the source, then the structure can be protected from seismic wave energy. Seismic metamaterials, regarded as periodic structures with properties different from conventional materials, use wave propagation characteristics and bandgaps to dissipate seismic wave energy. When the seismic wave is located in the bandgap, the transmission of seismic wave energy is effectively reduced, which protects the structure from the damage caused by seismic disturbance. In practical application, locating seismic frequencies below ten Hz is a challenge for seismic metamaterials. In the commonly used method, high-mass materials are employed to induce the effect of local resonance, which is not economically feasible. In this study, a lightweight design using auxetic geometry is proposed to facilitate the practical feasibility of seismic metamaterials. The benefits of this design are proven by comparing conventional seismic metamaterials with metamaterials of auxetic geometry. Different geometric parameters are defined using auxetic geometry to determine the structure with the best bandgap performance. Finite element simulations are conducted to evaluate the vibration reduction benefits of auxetic seismic metamaterials in time and frequency domains. Additionally, the relationship between the mass and stiffness of the unit structure is derived from the analytical solution of one-dimensional periodic structures, and modal analysis results of auxetic metamaterials are verified. This study provides seismic metamaterials that are lightweight, small in volume, and possess low-frequency bandgaps for practical applications.

## 1. Introduction

People living in the seismic belt are frequently threatened by earthquakes. The unpredictability of seismic activities makes residents bear the fear of losing life and property at any time. Therefore, seismic protection devices have been developed to protect structures from the huge energy propagated from the epicenter, which makes the structure much safer [1]. However, conventional energy dissipation elements such as dampers and bracing are typically installed on existing structures, inevitably affecting their dynamic parameters. In contrast, seismic metamaterials are a novel vibration mitigation method based on phononic crystal concepts that are not attached to the structure, thereby avoiding any impact on the dynamic characteristics of the structure and reducing surface disturbance. Consequently, seismic metamaterials can theoretically exhibit superior performance in attenuating dynamic propagation waves across a broad frequency range.

**Figure 1:**
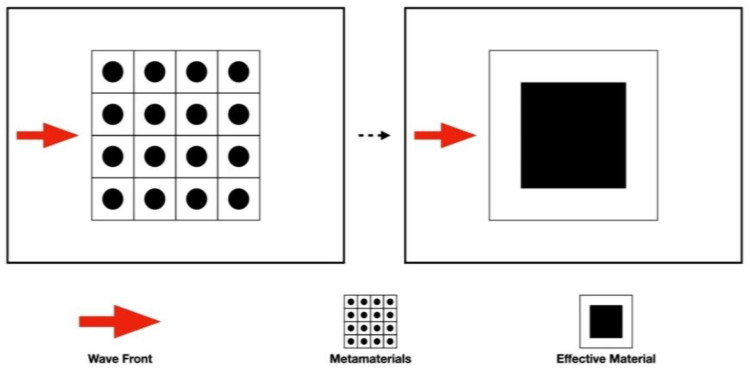
Schematic diagram of effective material.

In nature, unique behavior such as interference, diffraction, and scattering in periodic structures can be observed under the process of wave propagation. With proper design and arrangement, the phenomena of guiding, shielding, and filtering the specific frequency waves can be achieved [2,3,4]. The mechanism of the periodic structure with proper design can be simulated as an equivalent material which performs with the negative material property, as shown in Figure 1. This characteristic has been widely used in different fields [5,6]. Engineers usually relate negative stiffness with simple buckling-like effects in purely mechanical systems [7]. However, negative stiffness can be observed also in thermodynamic many-body systems [8]. For example, negative magnetic permeability and negative electric permittivity in the field of electromagnetism [9], negative stiffness and damping in the field of composite materials [10], negative density [11,12], and negative stiffness [13,14] in the field of mechanics can be achieved by designing the required periodic arrangement structure to obtain the expected phenomenon. In recent years, the concept of negative stiffness has been introduced in seismic isolation. In contrast to positive stiffness springs, true negative stiffness is defined as assisting motion rather than opposing it. It has been demonstrated that mechanical devices with negative stiffness significantly reduce the stiffness of the isolator and consequently reduce the natural frequency of the system even at almost zero levels [15]. In a broad sense, a novel material with different properties from conventional material is defined as a metamaterial.

The energy of an earthquake is propagated by an elastic wave, which can be classified as a mechanical wave that requires a medium for transmission [16,17,18,19]. In 1968, Woods et al. used vibrators to create shock waves to prove that elastic waves have similar behavior to electromagnetic waves [20]. Through periodic cavities and grooves, the surface displacement response of shock waves with a frequency content of 100–200 Hz can be effectively reduced. This phenomenon, which enables elastic waves of a specific frequency to be rapidly attenuated or terminated, is defined as a bandgap. The frequency of seismic waves is mostly located between 0.1 and 10 Hz. Up to the present, many methods have been proposed to allocate the bandgap frequency. Achaoui et al. [21] proposed that, by periodically arranging rows of piles inserted into the rock, an ultra-wide bandgap near 0 Hz can be created. However, as the row piles need to be inserted into the bedrock, the implementation in practice becomes a difficult issue.

Zhang et al. [22] discussed the effect of different types of steel pile sections and soil parameters on bandgap. The bandwidth can be widened by increasing the thickness of the flange plate, the width of the web plate, and the elastic modulus of the soil. However, an increase in soil density may reduce the bandwidth. Du et al. [23] proposed to use of soil as the matrix and adopted a seismic metamaterial composed of concrete and steel where the lower bandgap can reach 5.5 Hz. Chen et al. [24] proposed a seismic metamaterial whereby rows of circular piles on a concrete foundation with a resonant cylinder achieved a bandgap frequency of 1 to 9 Hz. The proposed metamaterials were arranged periodically to filter elastic waves in specific frequency bands. However, the construction cost was still too high, which means it is not feasible in practice. To achieve regional protection, using materials in large quantities is impractical. Some metamaterial studies switched to the consideration of mechanisms other than local resonance. The first mechanical model of auxetic structure based on geometry was proposed in 1985 by Almgren [25], and the first thermodynamically model built of purely geometrical molecules (hard cyclic hexamers), which spontaneously form an auxetic structure (a thermodynamically stable phase), was studied by Wojciechowski et al. [26,27,28]. Structures with auxetic properties exhibit unusual behavior under static and dynamic loads. For example, Michalski and Strek [29] proposed blast-resistant auxetic and non-auxetic sandwich plates using the finite element method. The results indicated that the auxetic plate had superior blast resistance when compared with the regular sandwich panel. Energy can be absorbed through the deployment of the auxetic layer. Ungureanu et al. [30] proposed a row of piles in an auxetic geometry which was placed under a structure to generate a bandgap of 33–47 Hz. Li et al. [31] proposed a three-dimensional negative-Poisson-ratio metal metamaterial lattice structure by adding a star structure to the traditional 3D concave structure. This research involved numerical simulations of mechanical properties and experimental models conducted with selective laser melting (3D printing). The experimental results were consistent with the changing trend in finite element simulation. Recently, at the macroscopic level and bandgaps, some purely geometrical auxetic model structures were studied [32,33,34]. The frequency of seismic waves is mostly located between 0.1 and 10 Hz; therefore, this paper proposes a lightweight design using metamaterials of auxetic geometry to save material consumption, further lower the bandgap frequency, and absorb the energy caused by the seismic waves.

The remainder of this paper is organized as follows. The methodology of auxetic materials, the Brillouin zone, and the principles of the dispersion curve are described in Section 2. The benefit of geometry optimization and the mechanism of bandgap frequency are presented in Section 3. The potential parameters are derived by a one-dimensional discrete model, which further decreases the bandgap frequency to fit the seismic frequency. Finally, a summary and the conclusion are provided in Section 4.

## 2. Methodology

The properties and formation of auxetics are first explained in this section. The Brillouin zone, which was the result of the earliest analysis of periodic structures, was proposed by the crystallographer Brillouin [35]. Due to the periodicity, the wave propagation behavior of the crystal can be obtained by analyzing only one unit of the structure. As the wave of crystal is transmitted by the electromagnetic wave, and the seismic waves are elastic waves, the principle of dispersion curves of the elastic wave is also explained.

### 2.1. Auxetics

Conventional materials such as concrete, steel, and rubber, when subjected to tensile (compressive) force in the axial direction, shrink (expand) laterally due to their tendency to maintain a constant volume. This phenomenon is well known as Poisson’s effect [36,37]. The definition of Poisson’s ratio is shown in Equation (1).

(1) $$\nu_{yx}=-\frac{\in_{x}}{\in_{y}}$$

where ∈*_x_* is the lateral strain; ∈*_y_* is the axial strain; *ν_yx_* is the Poisson’s ratio for loading in the *y*-direction.

**Figure 2:**
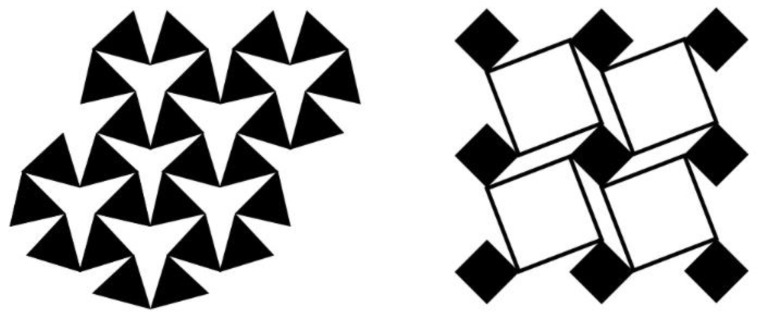
Schematic of rotating auxetic.

**Figure 3:**
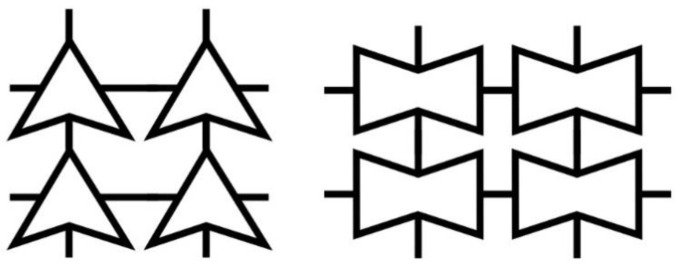
Schematic diagram of re-entrant auxetic.

Auxetics exhibit the opposite behavior to the conventional material. A material can be determined as auxetic if the Poisson’s ratio is less than zero. When the auxetics are under tension, the total material volume is increased by lateral expansion. Auxetics exist in nature in crystal scales such as pyrite crystals [37,38], which are hardly found in the field of civil engineering. However, by appropriate periodic arrangement or connection, the auxetic effect of the overall structure can be exhibited by utilizing regular materials. These developed mechanisms can be mainly divided into two types. For the first type, the auxetic effect is induced by rotation, as shown in Figure 2 [39,40]. With proper connection, the solid geometry in the figure behaves as a rigid body, which rotates under tension to show the auxetic effect. Secondly, re-entrant material is designed for the auxetic effect, as shown in Figure 3. When under tension, a large deformation is generated by this mechanism to achieve the auxetic effect. In this paper, the re-entrant auxetic mechanism is selected for lightweight design in practical application.

### 2.2. Brillouin Zone

The dispersion curve of the periodic structure is drawn by sweeping the frequency of the unit cell. The overall wave propagation behavior of periodic structures can be obtained by analyzing the unit cell, which is based on the theory of the first Brillouin zone. In this study, the reciprocal lattice space is adopted for the analysis in the Brillouin zone, where the lattice is the real spatial structure of the crystal. The real lattice space *R* can be defined as:

(2) $$R=m_{1}a_{1}+m_{2}a+m_{3}a_{3}$$

where $a_{1}$, $a_{2}$, and $a_{3}$ are unit lattice vectors; $m_{1}$, $m_{2}$, and $m_{3}$ are arbitrary integers.

The reciprocal lattice space *G*, which is similar to the definition of the lattice space, can be defined as:

(3) $$G=n_{1}b_{1}+n_{2}b_{2}+n_{3}b_{3}$$

where $b_{1}$, $b_{2}$, and $b_{3}$ are unit reciprocal lattice vectors; $n_{1}$, $n_{2}$, and $n_{3}$ are integers.

As the objective is a periodic structure, its boundary conditions are also set periodically as:

(4) $$\gamma_{i}(x)=\gamma_{i}(x+R)$$

where $\gamma_{i}$ is the displacement of the *i*th unit cell. The plane wave expansion method is applied to find the reciprocal lattice space as:

(5) $$P(\gamma )=Ce^{iG\cdot r}$$

where *C* is the lattice space; *r* corresponds to the displacement of the reciprocal lattice space; *P* is the reciprocal space mode.

By substituting the boundary conditions (Equation (6)) into Equation (5), the reciprocal space mode can be deduced as:

(6) P(r)⇒P(r+R)

(7) $$P(r+R)=Ce^{iG\cdot r}e^{iG\cdot R}$$

Moreover, the lattice unit vector and the reciprocal lattice unit vector are orthogonal as:

(8) $$a_{i}\cdot b_{i}=2\pi \delta_{ij}$$

where $\delta_{ij}=1,i=j{;\delta }_{\mathrm{ij}}=0,i\neq j$.

**Figure 4:**
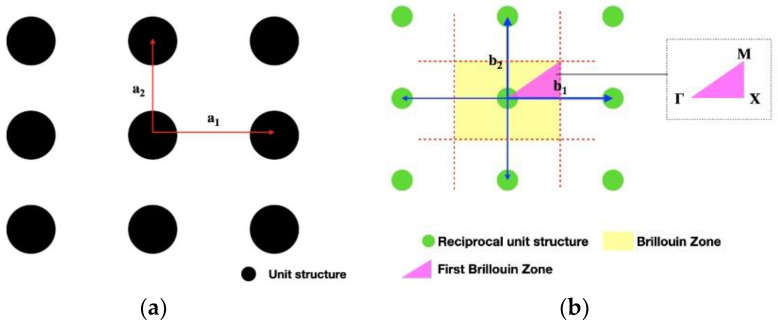
Schematic diagram of lattice and reciprocal lattice structure: (**a**) lattice structure; (**b**) reciprocal lattice structure.

Figure 4a,b shows a lattice and reciprocal lattice structure, respectively. After selecting a unit cell in the reciprocal lattice structure as the center, lines are connected to the surrounding unit cell by a blue line, as shown in Figure 4b. Subsequently, a perpendicular bisector of the blue line is drawn as a red dotted line, and the yellow area where it intersects is called the Brillouin zone. As the unit cell is periodic, the Brillouin zone can be further reduced to the first Brillouin zone, as shown by the magenta region in Figure 4. That is, by analyzing the first Brillouin zone, the wave propagation behavior of the whole structure can be known. The frequency sweep analysis of the two-dimensional square lattice can be conducted by sequentially replacing *k_x_* (wave number in the *x*-direction) and *k_y_* (wave number in the *y*-direction) into specific path points Γ (center of the Brillouin zone), X (center of the face), M (center of the edge), and Γ to obtain the bandgap characteristics of the two-dimensional square lattice.

### 2.3. Dispersion Curve

The bandgap characteristics of the periodic structure can be obtained by analyzing the first Brillouin zone of the unit cell. However, an alternative approach in the electromagnetic wave and the elastic wave domains is used to generate the dispersion curve. The three-dimensional wave propagation equation can be expressed as:

(9) $$-\rho \omega^{2}u=\frac{E}{2(1+\nu )}\nabla^{2}u+\frac{E}{2(1+\nu )(1-2\nu )}\nabla (\nabla \cdot u)$$

where *E* is the Young’s modulus of the material; *ν* is the Poisson’s ratio of the material; *u* is the displacement matrix; *ρ* is the density of the material; *ω* is the resonance frequency.

Due to periodicity, the boundary conditions can be expressed as:

(10) $$u(r+a)=e^{ik\cdot a}u(r)$$

where *k* is the Bloch wave vector; *r* is the position vector; *a* is the lattice vector.

By substituting Equation (10) into Equation (9), the eigenfunction can be derived as:

(11) $$(K-\omega^{2}M)U=0$$

where *K* is the stiffness matrix function of wave vector *k*; *M* is the mass matrix; *U* is the displacement matrix.

By substituting the wave vector k of the first Brillouin zone into the stiffness matrix in Equation (11), the eigenvalues can be obtained, and the elastic wave dispersion properties of this periodic structured lattice can then be generated. This supports another mechanism of generating the elastic wave bandgap.

## 3. Unit Cell

The unit cell of auxetic seismic metamaterials is introduced in this section. A unit structure is first designed, and the effect on the bandgap is evaluated by performing parametric analysis. Nevertheless, the bandgap frequency is still too high for earthquake protection. As a result, the one-dimensional periodic discrete model is derived with new parameters to further reduce the bandgap frequency.

### 3.1. Auxetic Metamaterial Unit

**Figure 5:**
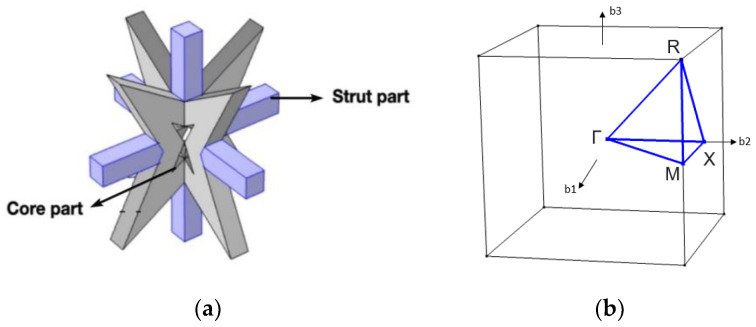
Schematic diagram of square lattice: (**a**) unit cell; (**b**) first Brillouin zone [41].

Due to the geometric complexity of auxetic metamaterials, a unit cell is divided into two parts to clarify its behavior, as shown in Figure 5a. One is the core part, and the other is the strut part, which connects each core part. The upper bound of the bandgap is determined by the strut part, and the lower bound of the bandgap is determined by the core part. The lattice selected for the analysis of the bandgap is a three-dimensional square lattice. The lattice constant of the auxetic metamaterial is arranged periodically and set to 10 m. Considering the feasibility of construction, the material is selected to be concrete, and its material coefficient is shown in Table 1. The frequency sweep of the first Brillouin zone is shown in Figure 5b, and the frequency sweep sequence is Γ, X, M, R (corner point), Γ.

### 3.2. Geometry Optimization of the Unit Cell

**Figure 6:**
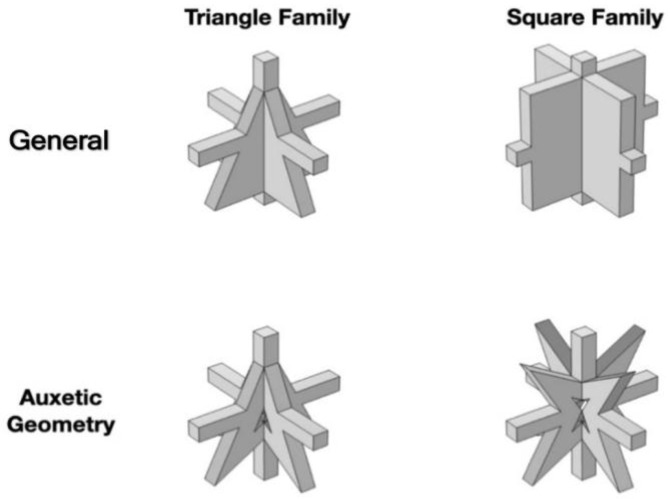
Schematic diagram of the unit cell.

**Figure 7:**
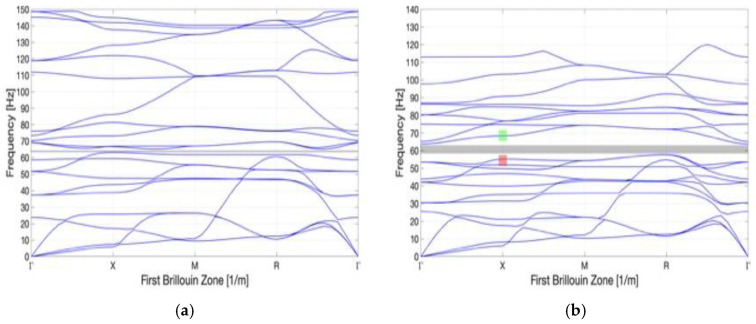

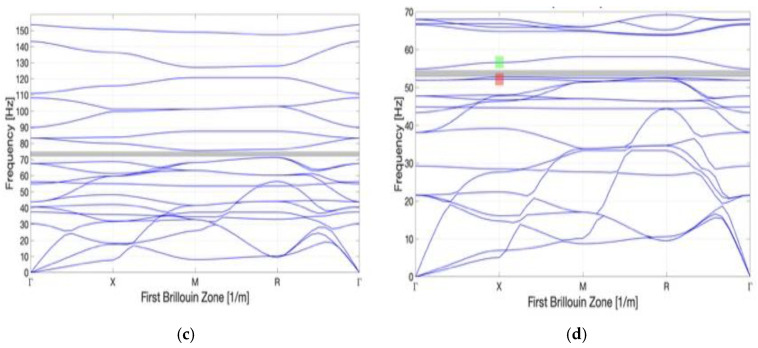
Dispersion curve for the triangle and square family: (**a**) general triangle; (**b**) auxetic geometry triangle; (**c**) general square; and (**d**) auxetic geometry square. The red shading region is the lower boundary bandgap mode, and the green shading region is the upper boundary bandgap mode.

To optimize the geometry of the unit cell, the core part is selected from a triangular family and a square family based on the cross-section geometry. As shown in Figure 6, each family is divided into general and auxetic geometry. By analyzing the bandgap characteristics of each family, it is found that better bandgap behavior can be performed by the auxetic geometry than the general one (Figure 7a–d). Table 2 shows the bandgap frequencies of each family. The general geometry of the triangular family has a bandgap frequency between 66.8 and 73.3 Hz with a bandwidth of 3.4 Hz. In contrast, the auxetic geometry of the triangular family has a bandgap frequency of 57.8–63.6 Hz and a bandwidth of 5.8 Hz. Meanwhile, the general geometry of the square family has a bandgap frequency of 71.3–76.3 Hz and a bandwidth of 5 Hz. Contrary, the auxetic geometry of the square family has a bandgap frequency of 51.9–54.9 Hz and a bandwidth of 3 Hz. It is observed that a lower bandgap frequency and lower material consumption can be reached by the auxetic metamaterial with proper cutting. The capability of both structures to absorb the energy is determined by the bandgap. According to the numerical simulation, the Poisson’s ratio is between −0.05 and −0.25, which varies with the strain when Equation (1) is applied. It is observed from the analysis that the values of Poisson’s ratio vary from −0.05 to −0.22 and −0.08 to −0.25 for the triangular and square shapes, respectively. Thus, the square metamaterial possesses a more negative Poisson’s ratio than the triangular one.

**Figure 8:**
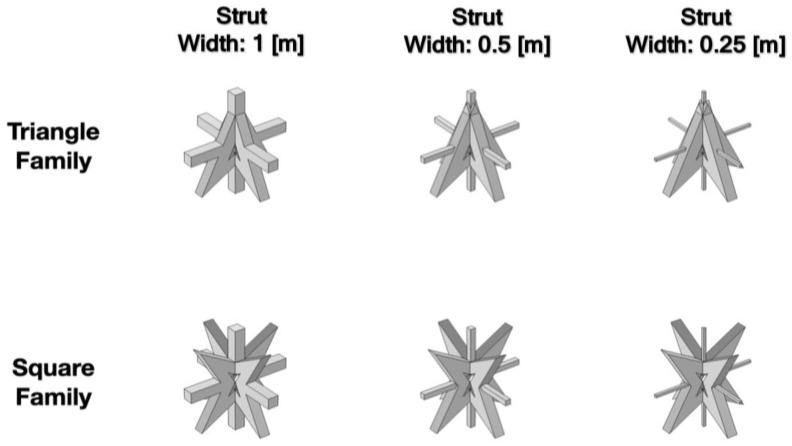
Schematic diagram of the unit cell with changing strut cross-section.

**Figure 9:**
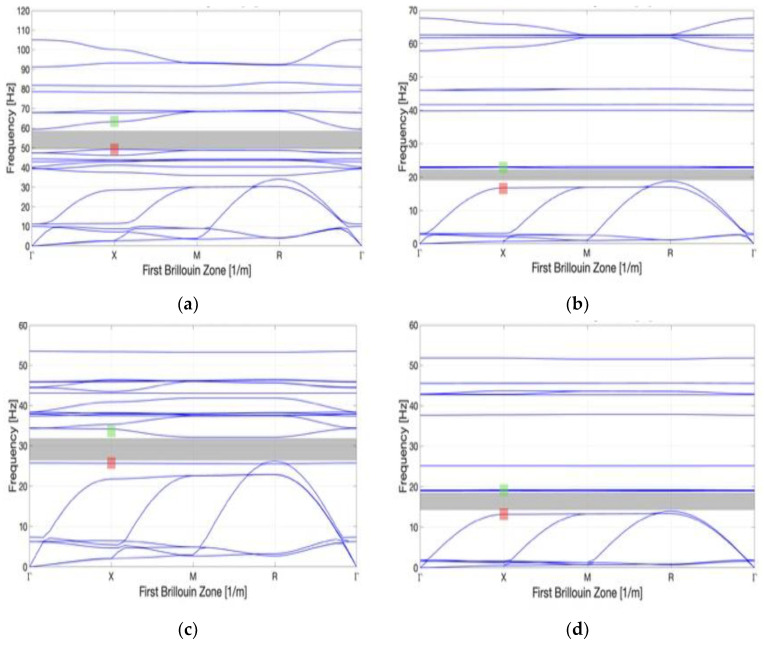
Dispersion curve of different widths: (**a**) triangle with strut width 0.5 m; (**b**) triangle with strut width 0.25 m; (**c**) square with strut width 0.5 m; and (**d**) square with strut width 0.25 m. The red shading region is the lower boundary bandgap mode, and the green shading region is the upper boundary bandgap mode.

The effect of the strut cross-section on the bandgap frequency is further explored. Based on the original model with a width of 1 m, the strut models with a width of 1 m, 0.5 m, and 0.25 m for each family shown in Figure 8 are discussed. The volume fraction of each strut model can be roughly estimated as 3%, 0.75%, and 0.188% based on the original unit cell, respectively. Bandgap frequencies of 48.9~59.4 and 19.1~22.4 Hz (bandwidths of 3.3 and 9.5 Hz) are possessed by the triangular family of auxetic geometry with a width of 0.5 m and 0.25 m, respectively. Similarly, the square family of auxetic geometry with a width of 0.5 m and 0.25 m has bandgap frequencies of 26.4~31.3 and 13.9~18.9 Hz, (bandwidths of 4.9 and 5 Hz), respectively. The dispersion curves and bandgap characteristics of these families are shown in Figure 9a–d and Table 3.

**Figure 10:**
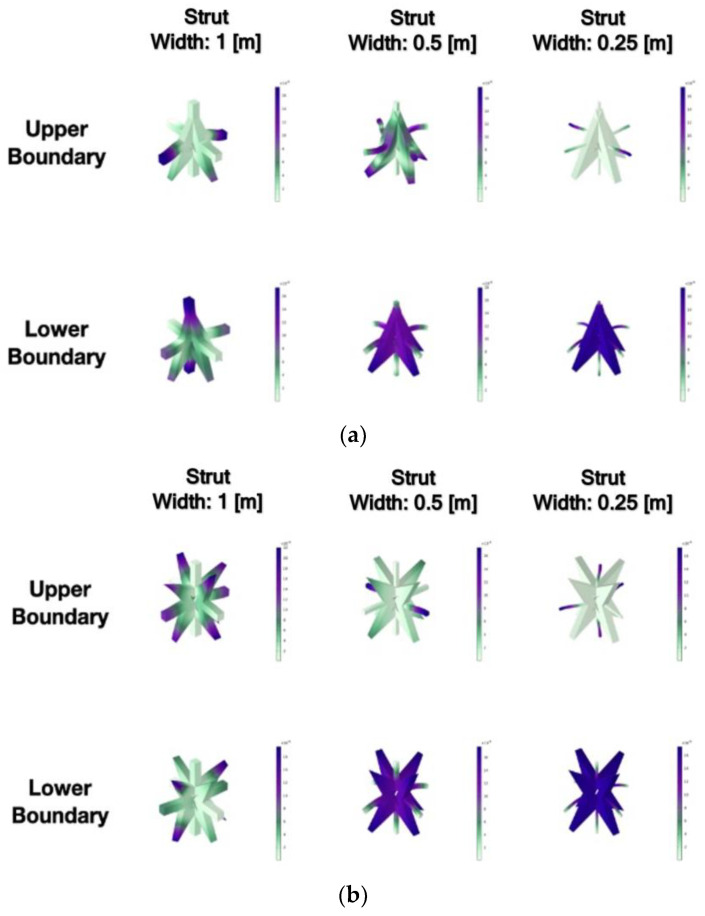
Upper and lower boundary modes of different geometric structures: (**a**) triangle family; (**b**) square family.

The mechanism for generating the bandgap is investigated through modal analysis, which allows the identification of modes corresponding to the upper and lower bounds of the bandgap. By reducing the width of the strut in the square of auxetic geometry, it is observed that the bandwidth does not decrease and even becomes wider. Figure 10 illustrates the modes associated with the upper and lower bounds of the bandgap for each geometry family. The intensity of color in the figure represents the magnitude of displacement in the local structure. The analysis results indicate that the strut component displays the highest vibrational energy and displacement within the upper bound bandgap mode. In contrast, the geometric core exhibits minimal displacement response in this mode. The lower bandgap mode is determined by the geometric core showing rigid body motion accompanied by consistent global displacement.

Moreover, in the upper bound bandgap mode, only the displacement of the strut with a width of 1 m is more severe, and the struts with a width of 0.5 m and 0.25 m have no significant displacement. However, the corners of the flanges of the core exhibit large local vibrational behavior. It is inferred that the upper and lower bound bandgap frequencies of the metamaterials of auxetic geometry are controlled by different factors. The upper bound bandgap frequency is mainly controlled by the vibration of the strut part of the structure, and the lower bound bandgap frequency is mainly controlled by the rigid body motion of the core structure. To generate an efficient bandgap, the vibration frequency of the strut must not be close to the rigid body motion frequency of the core structure. For the square family, the bandwidth is mainly caused by its core geometry, which is highly symmetrical and generates an auxetic effect. Because of the geometric symmetry, the dispersion curve in a single direction is not higher than the bandgap in the dispersion curve. Therefore, the square family can lower the bandgap frequency by reducing the width of the section and still retain a wider bandwidth.

### 3.3. One-Dimensional Discrete Periodic Model

Since the lowest bandgap frequency of metamaterials of auxetic geometry is still higher than 10 Hz, which is too high for earthquake protection, a one-dimensional model is adopted to further reduce the bandgap frequency.

**Figure 11:**
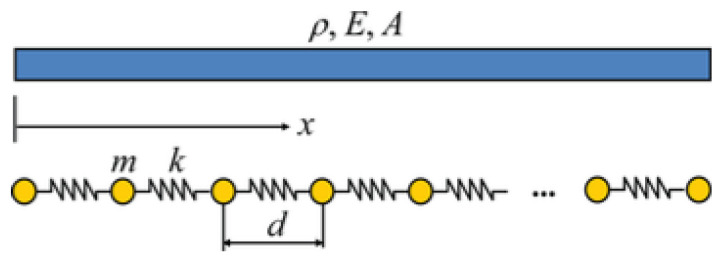
One-dimensional discrete periodic structure model.

The one-dimensional discrete periodic model shown in Figure 11 is reviewed [42,43,44]. A continuum beam member is discretized to simulate a mass point *m* and a concentrated stiffness *s* with a single degree of freedom. The equation of motion of each particle is shown as:

(12) $$m{\ddot{u}}_{n}+2su-s(u_{n-1}+u_{n+1})=0$$

where *m* is the mass of the discretized particle; *ü_n_* is the acceleration of the *n*th particle; *s* is the concentrated stiffness; *u_n_* is the displacement of the *n*th particle.

By the plane wave expansion method, the displacement *u_n_*(*t*) of the *n*th particle can be expressed through Equations (13)–(15).

(13) $$u_{n}(t)={\hat{u}}_{n}(\omega )e^{-i\omega t}$$

(14) $${\hat{u}}_{n}\left(\omega \right)=\tilde{u}\left(\mu \left(\omega \right)\right)e^{i\mu n}$$

(15) μ=ka

where the amplitude *û_n_*(*ω*) is the term related to the new variable *μ*, which is the product of the cycle number *k* and the lattice constant *a*; *ũ* is the arbitrary function for the plane wave expansion.

Substituting Equation (14) into Equation (12), a new eigenfunction can be derived as:

(16) $$-\omega^{2}m+2s\left(1-\cos\mu \right)=0$$

where *ω* is the frequency of the input disturbance.

**Figure 12:**
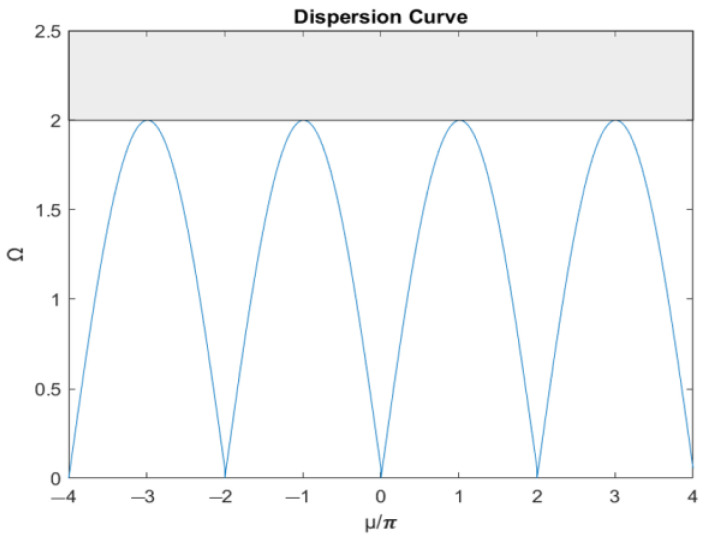
Dispersion curve by plane wave expansion method.

Equation (16) is the dispersion equation of the one-dimensional periodic structure, which describes the relationship between the disturbance frequency and the number of cycles. Taking u/π as the horizontal axis and *Ω* as the vertical axis, the dispersion curve can be depicted as shown in Figure 12, where *Ω* is the normalization of the disturbance frequency *ω* to the natural frequency *ω_n_* (*Ω* = *ω*/*ω_n_*).

The dispersion curve has a certain periodicity with a basic period between −1 and 1, which is the first Brillouin zone of a one-dimensional periodic structure. Because the disturbance is not always less than twice the natural frequency, the augmented plane wave method is used to further invert the relationship between the number of cycles and the frequency when the disturbance frequency is more than double. The augmented plane wave method is applied to solve the relationship between the number of cycles and the frequency as follows:

(17) $$\mu =\cos^{-1}\left(1-\frac{\Omega^{2}}{2}\right)$$

**Figure 13:**
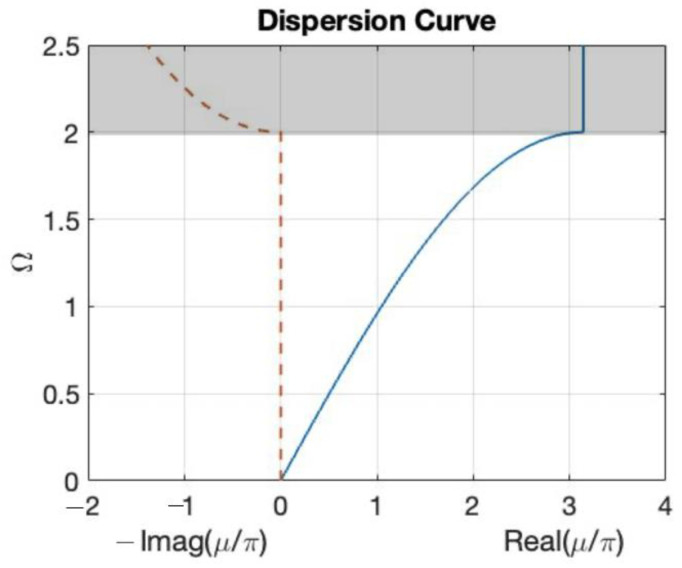
Dispersion curve by augmented plane wave expansion method.

As shown in the dispersion curve shown in Figure 13, the number of cycles is a complex number if the input external force frequency is higher than twice the natural frequency. In addition, as the frequency increases, the imaginary part also increases exponentially; that is, when the input external force is higher than twice the natural frequency, the energy propagation is dissipated exponentially with the spatial distribution. Therefore, a bandgap higher than twice the natural frequency can be generated by the one-dimensional periodic model as:

(18) Ω>2

The relationship between frequency and system parameters (mass, stiffness) can be further derived from Equation (18) as follows:

(19) $$m>\frac{s}{\pi^{2}f^{2}}$$

where *f* is the perturbation frequency.

Equation (19) also explains why conventional seismic metamaterials often use high-density steel to fabricate element structures: due to the low frequency of the seismic wave. For example, when the main frequency of the seismic wave is 0.1 Hz, the relation of its system parameters can be expressed as:

(20) $$m>\frac{s}{\pi^{2}0.1^{2}}\approx 10s$$

In summary, the mass of the system must be designed to be ten times higher than the system stiffness to fit the requirement of the bandgap.

### 3.4. Optimization of the Auxetic Geometry Unit

**Figure 14:**
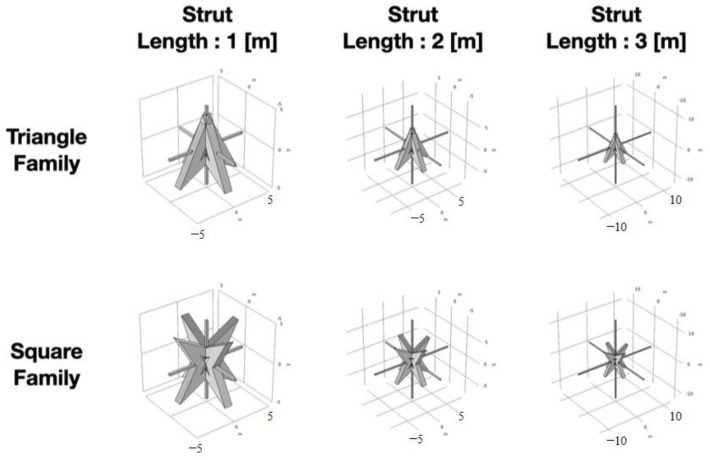
Schematic diagram of the unit cell with changing strut length.

Since the directional factor is not considered, the geometric–auxetic metamaterial can be simulated by a one-dimensional periodic model. The core part of the structure is regarded as the particle of the one-dimensional periodic model, and the strut part is regarded as the concentrated stiffness of the periodic structure. It is deduced that, if the stiffness can be effectively reduced, it does not need a huge mass to have bandgap characteristics. Assuming that the axial stiffness is provided by the strut, the axial stiffness shown in Section 3.2 can be adjusted by reducing the width of the section. Therefore, the mass of the core part remains unchanged, and the bandgap frequency can also be effectively reduced. In this section, the strut length is changed to study the effect of its stiffness on bandgap frequency, as shown in Figure 14.

The core geometry of each family is the same as mentioned previously. The setting of the dispersion curve is to obtain 20 characteristic frequencies for each cycle number. With 1/12 the interval of the cycle number, the cycle number of the loop swept by the finite element software is set to 4 to complete the definition of the Brillouin zone.

**Figure 15:**
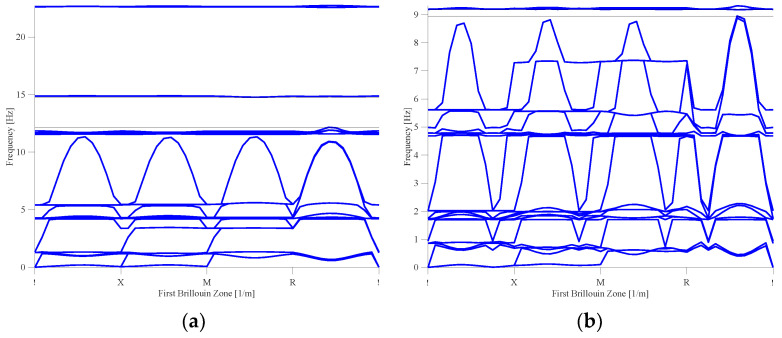

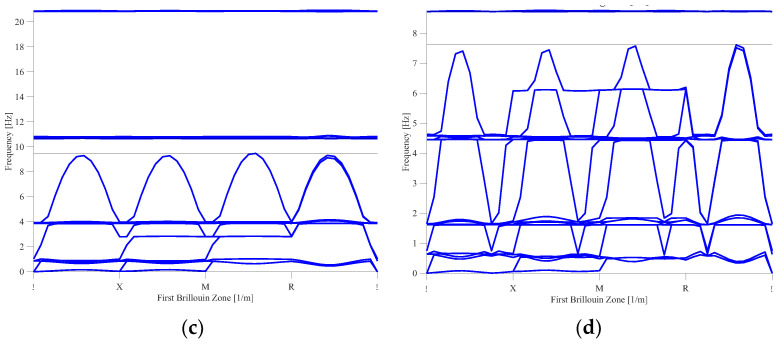
Dispersion curve of different lengths: (**a**) triangle with strut length 2 m; (**b**) triangle with strut length 3 m; (**c**) square with strut length 2 m; and (**d**) square with strut length 3 m.

Figure 15a,b shows the optimized geometric dispersion curves of the triangular family. The increasing strut length reduces the lower bound bandgap frequency from 19.1 Hz to 9 Hz, but the bandwidth is decreased from 3.3 Hz to 0.2 Hz. Figure 15c,d shows the optimized geometric dispersion curves of the square family. The increasing support length reduces the lower bound bandgap frequency from 13.9 Hz to 7.6 Hz, but the bandwidth is decreased from 5 Hz to 1.1 Hz.

As indicated in Table 4, increasing the strut length can lower the bandgap frequency, but it also narrows the bandwidth. Reducing the stiffness of the strut can solve the difficulty of tuning the structural mass of the core part shown in Equation (19). Through the two proposed design steps, reducing the width and increasing the length of the strut, it is found that the square shape exhibits lowering of the bandgap frequency with enough bandwidth. Compared to the triangular shape, which possesses lower symmetry leading to inferior results in reducing the bandgap frequency, the square shape achieves the target of this study to absorb the seismic energy. This conclusion provides a clear design guideline for metamaterials of auxetic geometry and suggests using the auxetic square metamaterial shape in practical applications to lower the bandgap frequency.

## 4. Summaries and Conclusions

This paper demonstrates that the bandgap performance of metamaterials of auxetic geometry, which have been widely studied for their special properties linked to a negative Poisson’s ratio *ν* [29,30], is superior to that of general metamaterial, both in terms of the amount of material used and the lower bound frequency of the bandgap. It is found that reducing the width of the strut section significantly decreases the lower bound frequency of the bandgap for all geometric shapes, but it also decreases the bandwidth. To enlarge the bandgap, it is necessary to adjust the relationship between the core mass and strut stiffness. The core structure needs to be driven to generate local resonance by reducing the strut stiffness with an auxetic effect. Modal analysis reveals that the generation of the bandgap mechanism requires a difference between the vibration frequency of the strut and the rigid body motion frequency of the core structure. The more significant the difference in vibration frequencies between the two components, the wider the bandgap that can be generated. The one-dimensional periodic structure study further confirms the results of the modal analysis. By considering the frequency range of the seismic waves, it is found that the strut stiffness needs to be reduced so that the core mass can easily generate an appropriate bandgap. The strut length is optimized to further reduce the lower bound frequency of the bandgap. The dispersion curve analysis demonstrates that changing the strut length can reduce the lower bound frequency of the bandgap to around 10 Hz.

This paper takes a geometric approach, complemented by a study of a one-dimensional discrete periodic structure, to offer alternative perspectives on developing bandgaps in seismic metamaterials. To locate the bandgap in the seismic zone, the system mass must be significantly larger than the system stiffness. The massive metamaterial approach suggested by previous research offers a solution; however, reducing the system stiffness can also be an effective method without the need for the massive metamaterial. This study demonstrates that square-shaped metamaterial exhibits good results in lowering the bandgap frequency and absorbing seismic energy after reducing the width and increasing the length of the strut, which meets the purpose of this study. Thus, it is suggested that using square-shaped auxetic metamaterial in practical applications can further lower the bandgap frequency. Furthermore, the performance of the proposed method can be verified by conducting experiments on metamaterials of auxetic geometry in future research.

## Figures and Tables

**Table 1:** Material parameters of the unit cell.

Material Parameters	Value
Density (kg/m^3^)	2400
Young’s modulus (GPa)	24.6
Poisson’s ratio	0.2

**Table 2:** Bandgap frequency.

Bandgap Properties (Hz)	Triangle Family		Square Family	
	General	Auxetic	General	Auxetic
Upper Boundary	73.3	63.6	76.3	54.9
Lower Boundary	66.8	57.8	71.3	51.9
Bandwidth	3.4	5.8	5	3
Poisson’s Ratio		−0.05~−0.22		−0.08~−0.25

**Table 3:** Bandgap frequency with changing strut cross-section.

Family Type	Triangle Family			Square Family		
Strut Width (m)	1	0.5	0.25	1	0.5	0.25
Upper Boundary (Hz)	63.6	59.4	22.4	54.9	31.3	18.9
Lower Boundary (Hz)	57.8	48.9	19.1	51.9	26.4	13.9

**Table 4:** Bandgap frequency with changing strut length.

Family Type	Triangle Family			Square Family		
Strut Width (m)	1	2	3	1	2	3
Upper Boundary (Hz)	22.4	14.5	9.2	18.9	10.6	8.7
Lower Boundary (Hz)	19.1	11.4	9	13.9	9.8	7.6
Bandwidth (Hz)	3.3	3.1	0.2	5	0.8	1.1

## Data Availability

The data presented in this study are available upon request from the corresponding author. The data are not publicly available due to privacy concerns.
